# Thoracic extradural malignant melanoma with unknown primary

**DOI:** 10.1259/bjrcr.20200042

**Published:** 2020-07-17

**Authors:** Yasutaka Takagi, Hiroshi Yamada, Hidehumi Ebara, Hiroyuki Hayashi, Satoshi Kidani, Shunro Okamoto, Kazu Toyooka, Kazuhiro Nanpo, Yoshiyuki Kitano, Shintaro Terahata, Hiroyuki Tsuchiya

**Affiliations:** 1 Department of Orthopaedic Surgery, Tonami General Hospital, 1-61 Shintomi-cho, Tonami City, Toyama, Japan; 2 Department of Diagnostic Pathology, Tonami General Hospital, 1-61 Shintomi-cho, Tonami City, Toyama, Japan; 3 Department of Orthopaedic Surgery, Graduate School of Medicine, Kanazawa University, 13-1 Takara-machi, Kanazawa City, Ishikawa 920-8641, Japan

## Abstract

Primary extradural spinal melanoma is a very rare lesion. Here, we report a thoracic extradural malignant melanoma in a 77-year-old male. MRI showed a dorsal spinal extradural tumour at the T6–T8 level. The tumour showed hyperintensity on T1W imaging and mixed hypointensity and hyperintensity on T2W imaging. Gadolinium-enhanced MRI showed high enhancement on the lesion. Information on imaging findings for extradural spinal melanoma appears very limited. We discuss the MRI findings from this case, which can be considered typical of extradural spinal melanoma and review the literature.

## Introduction

Primary extradural spinal melanoma is a very rare lesion, with only nine primary extradural spinal melanomas reported to date.^1–7^ Of these, four occurred in the spinal epidural space.^1,2,5,7^ We report a very rare case of primary thoracic extradural malignant melanoma.

## Clinical presentation

A 77-year-old male was admitted to our hospital with a history of progressive paraplegia and paresthesia for about 2 weeks. He had no medical history of note and was not on any medications. Neurological examination revealed spinal cord insufficiency paraplegia. Laboratory data were within normal limits.

## Investigations

**Figure 1.**
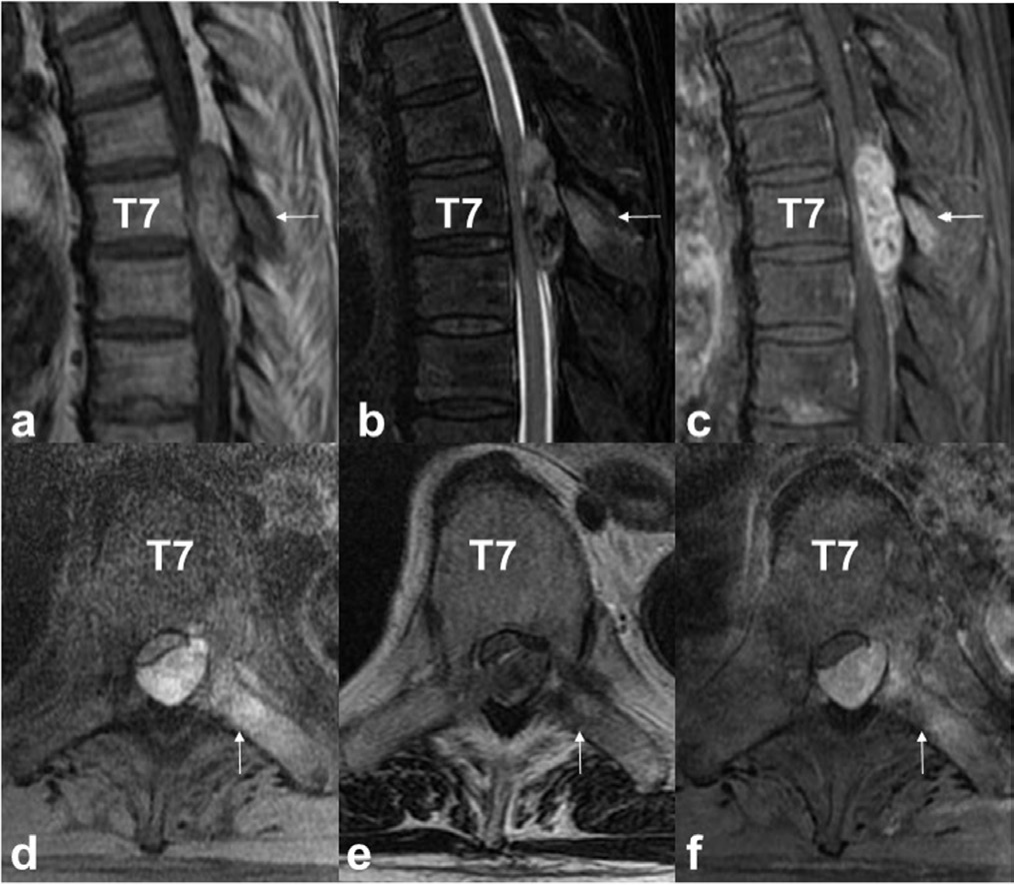
MRI shows a dorsal spinal extradural tumour of T6–T8. The tumour appears hyperintense on T1 (a: sagittal; d: axial, fat suppression) with mixed hypointensity and hyperintensity on T2 (b: sagittal; d: axial). Gadolinium-enhanced MRI shows high enhancement on the lesion (c: sagittal, fat suppression; f: axial, fat suppression). The T7 lamina and left transverse process show signal intensity changes (white arrow).

**Figure 2.**
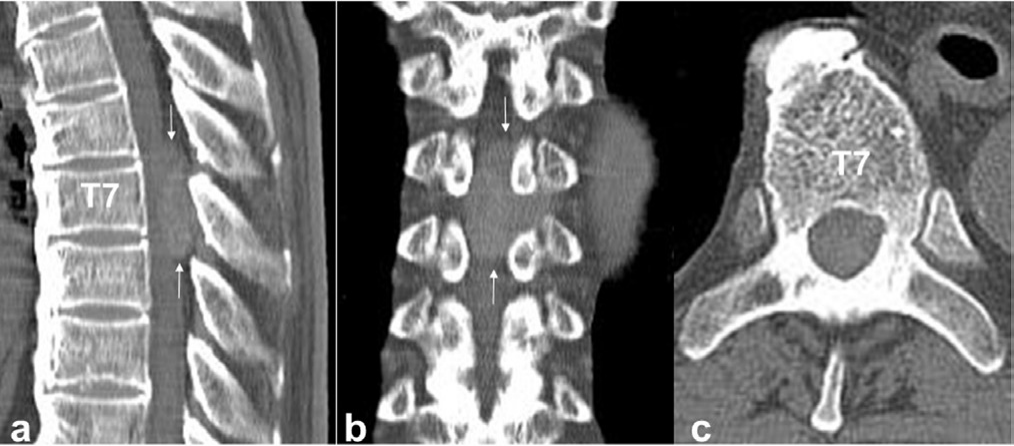
CT shows the T7 vertebral body, pedicle and lamina are free from osteolysis and other changes (a: sagittal; b: coronal; c: axial).

MRI showed a dorsal spinal extradural tumour at the T6–T8 level. The tumour appeared hyperintense on T1W imaging and showed mixed hypointensity and hyperintensity on T2W imaging. Gadolinium-enhanced MRI showed high enhancement on the lesion. The T7 lamina and left transverse process showed signal intensity changes (Figure 1). CT showed the T7 vertebral body, pedicle and lamina were free from osteolysis and other changes (Figure 2). For pathological diagnosis and to address spinal cord insufficiency paraplegia, the patient underwent surgery via a posterior approach.

## Treatment and outcome

**Figure 3.**
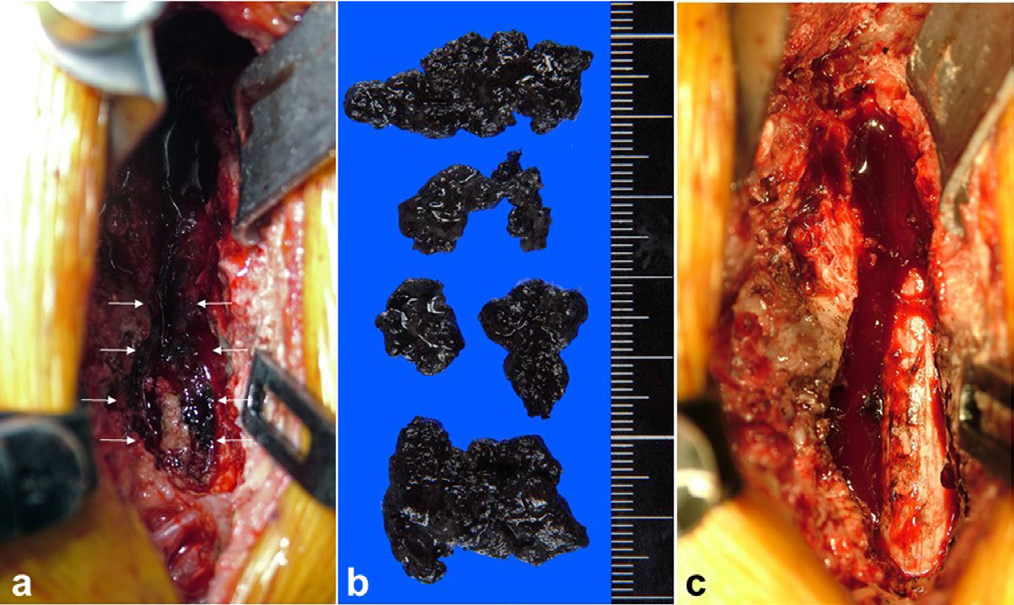
A black extradural tumour has invaded the lamina, spinous process and left transverse process of T7 (a) and resected tumour (b). The spinal cord is decompressed after tumour resection (c).

**Figure 4.**
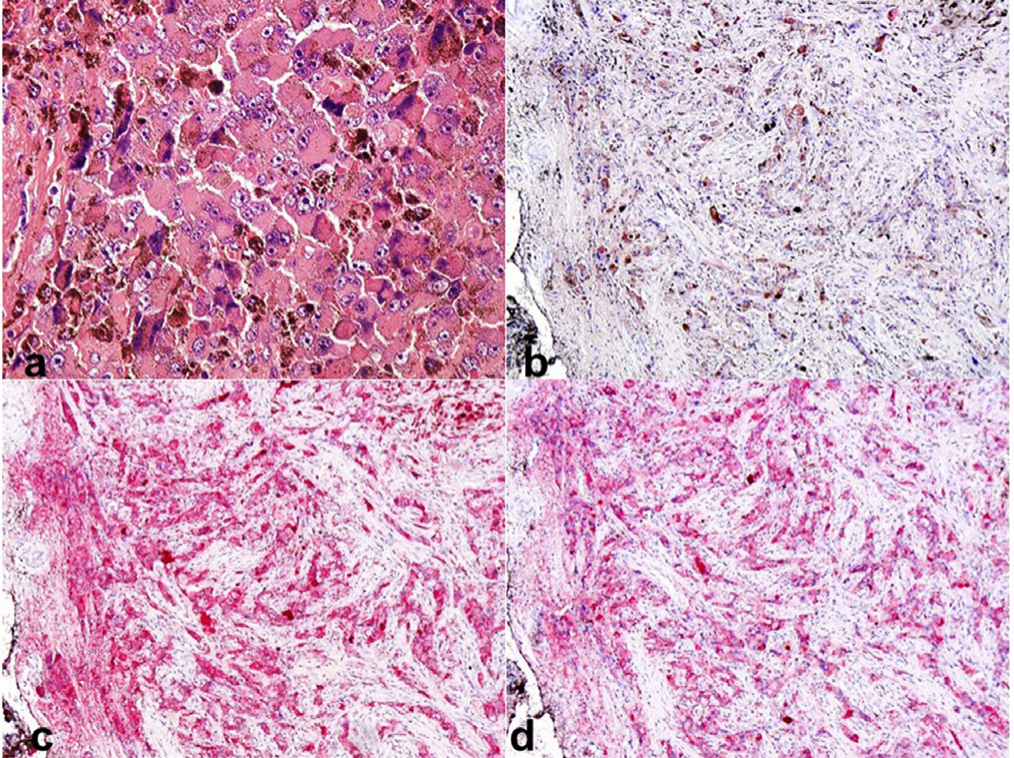
Pathological examination reveals diffuse proliferation of pigmented epithelioid tumour cells. Haematoxylin and eosin (a) (×400). Immunohistochemical staining using the labelling enzyme alkaline phosphatase and the chromogenic substrate new fuchsin confirms tumour cells are stained red for melanoma markers S-100 (b), HMB-45 (c), and Melan A (d), confirming tumour cells. (×20).

After T6–T8 laminectomy, a black extradural tumour was identified invading the lamina, spinous process and left transverse process of T7. The tumour was resected sufficiently and the spinal cord was decompressed. The tumour was identified as an extradural tumour in the operative field (Figure 3). Pathological examination of permanent sections revealed diffuse proliferation of pigmented epithelioid tumour cells. Immunohistochemical staining using the labelling enzyme alkaline phosphatase and the chromogenic substrate new fuchsin confirmed tumour cells stained red for melanoma markers S-100, HMB-45 and Melan A, confirming tumour cells (Figure 4). Based on these results, malignant melanoma was diagnosed. No primary malignant melanoma was identified from oncological and dermatological examinations. 16 days postoperatively, the patient underwent spinal radiotherapy to T5–T9, comprising 30 cGy in 10 fractions. After the procedure, neurological conditions were improved and he was able to walk using two 4-point canes. The tumour recurred 10 months postoperatively, and the patient died 1 year postoperatively, due to multiple metastases.

## Discussion

Most malignant melanomas that occur in the central nervous system (CNS) are metastatic,^1,2^^)^ and most spinal melanomas are intradural, with or without extradural components.^2^ Most melanomas have a known primary site, with only about 3.2% located in distant sites with an unknown primary site.^8^ Melanoma of unknown primary is most often located in the lymph nodes, followed by subcutaneous sites and finally the internal organs.^8^ Various hypotheses have been proposed regarding the origins of melanoma of unknown primary, including spontaneous regression of the primary tumour, and the presence of ectopic melanocytes in the lymph nodes and internal organs.^8^ The diagnosis of primary melanoma is based on absence of melanoma outside the CNS, the absence of melanoma in any other CNS sites, and histological confirmation of melanoma using the Hayward classification.^9^ This case met all these criteria and was diagnosed as primary melanoma.

MRI findings for melanoma vary significantly depending upon the number of melanin-containing cells and the amount of haemorrhage.^10,11^ In a typical melanotic melanoma, melanin has a paramagnetic effect due to the presence of stable organic radicals inside the tumour. The unpaired electrons of these free radicals interact with water protons, resulting in reduced relation times for both T1 and T2, producing hyperintensity on T1W imaging and hypointensity on T2W imaging. Meningioma, schwannoma and haemangiomatous lesions such as cavernous haemangioma or haemangiopericytoma are rarely purely extradural and may show intratumoural haemorrhage.^1,2,12^ Schwannomas and meningiomas are well-demarcated lesions that may also have melanin pigment and reveal hyperintensity on T1W imaging.^1,13^ Most spinal meningiomas are intradural, but King et al^12^ reported that about 2.5% are purely extradural. Another melanin-containing lesion, meningeal melanoma, is rare and may be indistinguishable from common melanoma in the cervical region.^14^

Primary malignant melanomas of the CNS have an equal sex ratio and generally occur in the fifth decade of life (age range, 20–80 years).^15^ Only nine primary extradural spinal melanomas have been reported to date.^1–7^ Of these, four occurred in the spinal epidural space.^1,2,5,7^ Jo et al.^2^ reported a case in which the mass was considered to be metastatic tumour based on the age of the patient and the MRI findings (epidural, highly enhanced, pedicle involvement). They searched for the primary tumour lesion using chest and abdominal CT, gastroscopy, colonoscopy, bone scan, tumour marker levels and mammography, but failed. In our case, the tumours were located in the extradural space, T7 lamina and left transverse process, with no primary malignant melanoma identified from oncological or dermatological examinations. Our case was thus diagnosed as a very rare malignant melanoma outside the dura mater.

The survival rate of patients with CNS melanoma is not known exactly, but the prognosis is better than that of patients with cutaneous melanoma.^5^ Complete surgical resection is the treatment of choice and radiotherapy is recommended for subtotal resection.^5^ Recently, immunomodulation with drugs such as ipilimumab and therapeutics targeting specific mutations such as the BRAF (v-raf murine sarcoma viral oncogene homolog B1) inhibitors vemurafenib and dabrafenib have achieved favourable increases in progression-free survival for patients with metastatic melanoma.^16^

## Learning points

1. We report a very rare case of primary thoracic extradural malignant melanoma.
2. Malignant melanoma should be suspected in extradural spinal cord tumours showing a characteristic MRI signal pattern of hyperintensity on T1W imaging and hypointensity on T2W imaging.
